# Harnessing prebiotic formamide chemistry: a novel platform for antiviral exploration

**DOI:** 10.1038/s41598-025-14001-3

**Published:** 2025-08-02

**Authors:** Maria Grazia Martina, Chiara Vagaggini, Elena Dreassi, Marta De Angelis, Lucia Nencioni, Filippo Dragoni, Federica Giammarino, Adele Boccuto, Maurizio Zazzi, Ilaria Vicenti, Marco Radi

**Affiliations:** 1 Dipartimento di Scienze degli Alimenti e del Farmaco, Università degli Studi di Parma, Viale delle Scienze, 27/A, Parma, 43124 Italy; 2 Department of Biotechnology, Chemistry and Pharmacy (DBCF), University of Siena, Siena, 53100 Italy; 3 Department of Public Health and Infectious Diseases, Laboratory Affiliated to Istituto Pasteur Italia-Fondazione Cenci Bolognetti, Sapienza University of Rome, P.le Aldo Moro 5, Rome, 00185 Italy; 4 Laboratory of Virology, Department of Molecular Medicine, Sapienza University, Rome, 00185 Italy; 5 Department of Medical Biotechnologies, University of Siena, Viale Bracci 16, Siena, 53100 Italy

**Keywords:** Prebiotic chemistry, Formamide model, Antiviral activity, Complexity, Co-evolution, Virology, Origin of life

## Abstract

Viruses and host cells are intricately connected through a shared “chemical language” that may trace back to the prebiotic chemistry of early Earth. In this study, we present an innovative platform for antiviral exploration inspired by this primordial chemical framework. By “doping” the formamide-based prebiotic chemistry model with orotic acid derivatives, we generated complex, non-natural chemical mixtures capable of disrupting the replication of multiple viruses with minimal or no toxicity for eukaryotic cells. This strategy underscores the potential of an evolution-inspired approach in antiviral discovery, offering a novel avenue for identifying new agents with unconventional mechanisms that might elude traditional discovery methods.

**Supplementary Information:**

The online version contains supplementary material available at 10.1038/s41598-025-14001-3.

## Introduction

Viruses and cells have been intricately linked since the dawn of life on Earth due to viruses’ reliance on host cells machinery for their survival. This relationship traces back to the origin of life on Earth, with all known life forms descending from a common ancestor, an organism often reported as Last Universal Common Ancestor (LUCA)^1^. The coevolution of LUCA and ancient viruses was driven by a shared biochemical machinery, exploiting a common “chemical language” for storing and transmitting genetic information as well as for expressing defensive and signaling compounds, which is based on molecular building blocks, such as nitrogenous bases, aminoacids, carbohydrates, and lipids. The generation of this common “chemical language” is directly linked to the assembly of simple inorganic molecules that were present in the prebiotic environment, which were transformed into primary and secondary heterocyclic metabolites (organic precursors) via Multi-Component Reactions (MCRs)^2,3^. MCRs generated awesome chemical diversity, setting the basis for the emergence of non-living matter and the evolution of all living organism on our planet. Among the key chemical precursors that could have served as starting materials of all life forms, hydrogen cyanide (HCN)^4^ and formamide (NH_2_CHO)^5,6^ have attracted growing attention as plausible building blocks for prebiotic MCRs. A prominent model proposes that formamide, generated by the hydrolysis of HCN, could have been accumulated in substantial amounts, serving as both a structural framework and reaction medium for the synthesis of essential biogenic molecules, such as nucleobases, acyclic nucleosides, nucleotides, carboxylic acids, sugars, amino sugars, amino acids, and condensing agents^7^. This formamide-based model offers a compelling explanation for the prebiotic synthesis of life’s first molecular components, as demonstrated by the work of Saladino and colleagues^8–10^.

In addition to chemical precursors, compartmentalized systems were also essential for the assembly of early life forms. Membrane-like structures, such as micelles and vesicles, were formed from amphiphilic molecules, creating suitable environments for chemical reactions to occur^11–13^. Several studies have explored how complex molecules, similar to phospholipids, could form abiotically from simpler amphiphilic molecules, proposed as potential prebiotic membrane lipids^14–18^. Fatty alcohols (long-chain alcohols with C4-C6) are considered potential components of the prebiotic environment that generated primordial membranes, while water served also as a catalyst of prebiotic reactions^19,20^. These molecules could play a significant role in the assembly of early life forms, highlighting their contribution to the origins of LUCA. From an evolutionary perspective, LUCA initially consisted of a minimal set of essential genes necessary for cellular life^21^ and, over time, evolved into a sophisticated machine equipped with a convoluted defence mechanism. In contrast, viruses have always been simple entities, unable to survive on their own but relying on host cell machinery for their survival and replication^22,23^.

By leveraging our current understanding of prebiotic chemistry, we hypothesized that the formamide-based prebiotic model could be modified in the lab to produce chemical mixtures that simulate microenvironments enriched with non-natural molecules—compounds that biological evolution would likely never have produced or selected. These novel chemical entities may represent an alternative “chemical language”: one that is tolerated or overlooked by the complex defence mechanisms of eukaryotic cells, yet remains undecipherable—and potentially disruptive—to simpler viral systems. This working hypothesis may offer a new conceptual framework for antiviral exploration, one that lies outside conventional evolutionary pathways and established drug discovery strategies.

In this study, we sought to translate our working hypothesis into a proof of concept by assessing the replication of various viruses in cells exposed to non-natural, evolution-inspired chemical mixtures. Our goal was to identify novel compounds with broad-spectrum antiviral potential. We finally demonstrated that formamide-based multicomponent reactions (MCRs), when carried out under modified, unconstrained conditions, can be directed toward unexplored regions of chemical space—producing complex mixtures that inhibit viral replication while exerting minimal or no cytotoxic effects on host cells. Although our approach is inspired by the formamide-based MCR model, it is not intended to replicate prebiotic conditions or simulate evolutionary processes. Rather, we use this chemistry as a conceptual platform for generating molecular diversity with potential antiviral activity.

## Results

### Design of the evolution-inspired approach

By investigating variables such as temperature, mineral surfaces, UV radiation, chemical environments, and atmospheric conditions, scientists have gained valuable insights into how life’s building blocks may have originated from inorganic precursors on early Earth^24^. Numerous studies across different laboratories have shown that formamide, under a variety of experimental conditions, can yield key biomolecules, including nucleic acids, amino acids, and lipids^25^. Elevated experimental temperatures, simulating conditions likely present on early Earth, not only facilitate the formation of nucleobases but also drive the synthesis of natural sugars and amino acids through multicomponent reactions (MCRs) involving formamide. In this work, we decided to repurpose this widely accepted formamide prebiotic model, redirecting it toward the generation of non-natural chemical mixtures with potential antiviral activity. The idea we enforced to achieve this goal was to add a “doping agent” to trigger MCRs that would be unlikely or inaccessible under traditional prebiotic conditions, simulated here by the formamide model. Specifically, we selected non-natural derivatives of orotic acid^26^ (compounds **1–3**) as doping agents, which are reactive analogues previously developed by our group as early intermediates in the synthesis of antiviral molecules (see Supplementary Material for synthetic details).

**Fig. 1:**
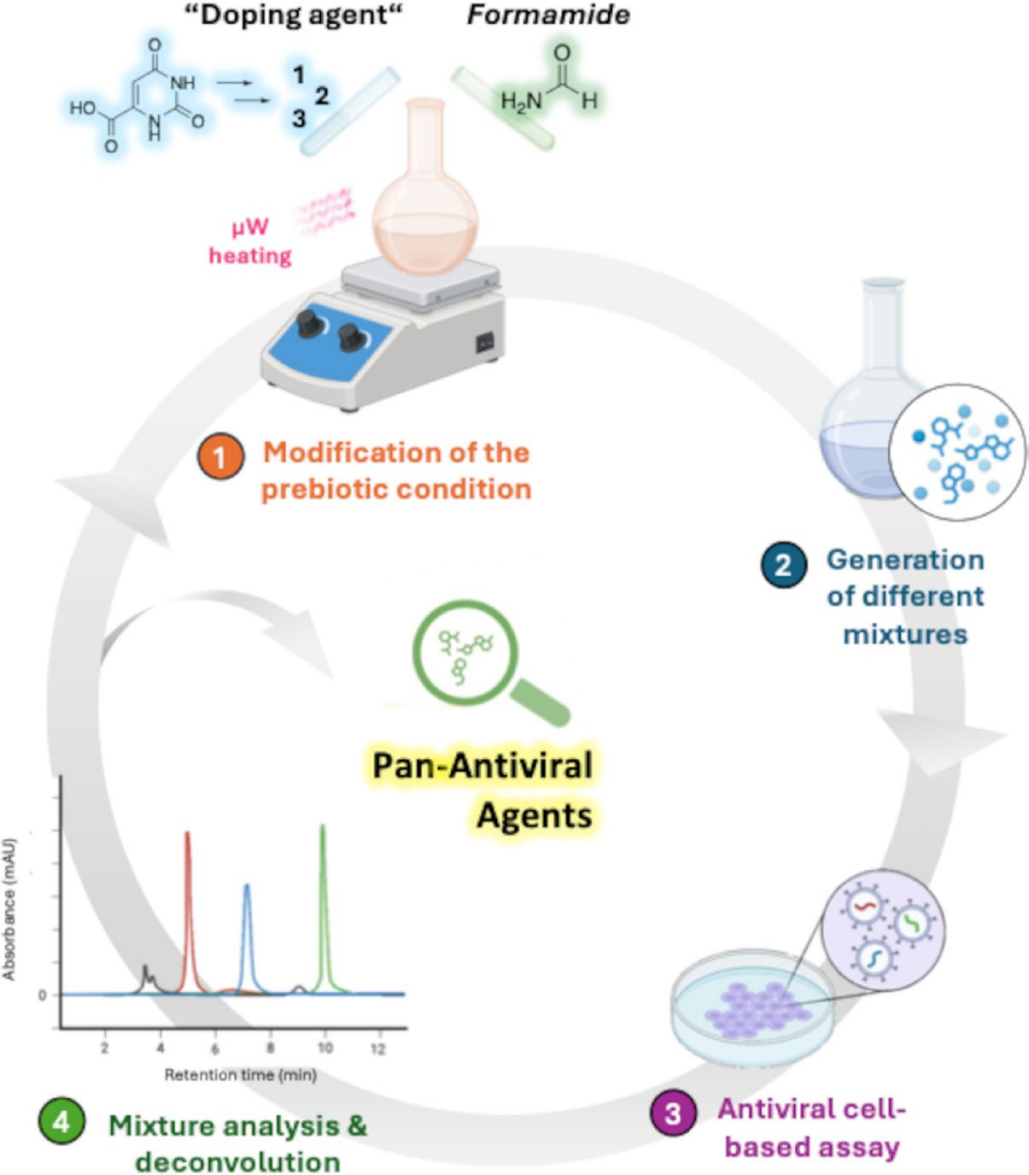
The four-step evolution-inspired approach for antiviral discovery.

The planned work (Fig. 1) follows a systematic four-step approach: (1) conducting the modified prebiotic reaction in the presence of different doping agents, using microwave irradiation as heating source; (2) isolating the mixtures of components soluble in a 90:10 EtOH: H₂O solution, representing molecules likely to be soluble in a prebiotic environment; (3) testing the isolated mixtures on cells infected with different viruses to select those with the most promising pan-antiviral properties; (4) analyzing and deconvoluting the selected active mixtures to identify active components and adjust the prebiotic conditions to improve the antiviral outcomes.

### Generation of non-natural prebiotic mixtures and antiviral screening

Our experiments were conducted in different solvents, such as water, 1:1 mixture pentanol: water, pure pentanol or pure formamide. The heating conditions reached 180 °C, for different time points (Table 1). We chose water and pentanol as solvents, considering their plausible presence on the prebiotic Earth, as mentioned in the previous section. In analogy with literature’s experiments based on the standard formamide prebiotic model, our modified protocol generated complex mixtures containing many different products. Both biological and analytical studies were conducted on those fractions of the prebiotic mixtures that were soluble in EtOH: H_2_O 90:10. This strategic choice was driven by the expectation that hydroalcoholic extracts would contain molecules with improved solubility for biological assays and be more amenable to streamlined chromatographic analysis. Thus, the mixtures obtained by modifying the prebiotic conditions were first treated with EtOH: H_2_O 90:10 solution. After thoroughly mixing, the resulting suspension was filtrated to separate the soluble fraction from the insoluble residue. The hydroalcoholic fractions (**Mix1 a-d**, **Mix2 a-d**, and **Mix3 a-d**), containing compounds more amenable to biological testing and chromatographic analysis, were then collected for further study.

**Table 1:** Synthesis of modified prebiotic mixtures **Mix1a-d**, **Mix2a-d**, and **Mix3a-d**.

Entry	Doping agent	Reagents and conditions	Products
1		NH_2_CHO (10 eq.), H_2_O 180 °C µW, 5 min	**Mix 1a**
2		NH_2_CHO (10 eq.), H_2_O/n-pentanol (1:1), 180 °C µW, 15 min	**Mix 1b**
3		NH_2_CHO (10 eq.), n-pentanol, 180 °C µW, 1 h	**Mix 1c**
4		NH_2_CHO (1 mL), 180 °C µW, 5 min	**Mix 1d**
5		NH_2_CHO (10 eq.), H_2_O 180 °C µW, 5 min	**Mix 2a**
6		NH_2_CHO (10 eq.), H_2_O/n-pentanol (1:1), 180 °C µW, 15 min	**Mix 2b**
7		NH_2_CHO (10 eq.), n-pentanol, 180 °C µW, 1 h	**Mix 2c**
8		NH_2_CHO (1 mL), 180 °C µW, 5 min	**Mix 2d**
9		NH_2_CHO (10 eq.), H_2_O/n-pentanol (1:1), 180 °C µW, 5 min	**Mix 3a**
10		NH_2_CHO (10 eq.), n-pentanol, 180 °C µW, 30 min	**Mix 3b**
11		NH_2_CHO (10 eq.), n-pentanol, 180 °C µW, 1 h	**Mix 3c**
12		NH_2_CHO (1 mL), 180 °C µW, 5 min	**Mix 3d**

Within these complex non-natural mixtures lies the potential for discovering innovative antiviral molecules not naturally selected by conventional processes. Thus, the next step consisted in testing these isolated mixtures in cells infected with different viruses, including West Nile virus (WNV), Dengue virus (DENV), and Retrovirus (HIV-1). As reported in Table 2, most mixtures exhibited minimal or no cytotoxicity in two different cell lines (Huh7 and H9). However, exceptions were noted, with **Mix 1b**, displaying low CC_50_ values in both cell lines. Notably, **Mix 1c** and **Mix 3c** turned out to be the most interesting in terms of antiviral activity: they inhibited, at low micromolar concentrations, the replication of both WNV and DENV with good selectivity index (SI).

**Table 2:** Cytotoxicity and antiviral activity of mixtures obtained through the modified prebiotic conditions.

Cmpd.	CC_50_^a^ (Huh7)	IC_50_^b^ WNV	IC_50_ DENV	CC_50_ (H9)	IC_50_ HIV
**Mix 1a**	40	NA^c^	NA	40	NA
**Mix 1b**	12	4.8 ± 3.8 (SI^d^ = 2.5)	1.9 ± 0.4 (SI = 9.4)	18	NA
**Mix 1c**	64	3.3 ± 1.4 (SI = 19.5)	7.2 ± 2.7 (SI = 8.8)	26	NA
**Mix 1d**	20	NA	NA	24	NA
**Mix 2a**	> 80	NA	NA	> 40	NA
**Mix2b**	> 80	NA	NA	> 40	NA
**Mix 2c**	56	NA	NA	40	6.2 ± 1.9 (SI = 6.4)
**Mix 2d**	> 80	NA	NA	> 40	NA
**Mix 3a**	24	8.0 ± 2.9 (SI = 3)	4.3 ± 1.9 (SI = 5.6)	18	NA
**Mix 3b**	> 80	4.3 ± 3.3 (SI > 18.7)	NA	48	NA
**Mix 3c**	> 80	3.2 ± 0.2 (SI > 25.3)	13.6 ± 2.7 (SI > 7.1)	40	NA
**Mix 3d**	72	NA	NA	40	NA

^*a*^*CC*_*50*_ half-maximal cytotoxic concentration (µg/mL), ^*b*^*IC*_*50*_ half-maximal inhibitory concentration (µg/mL), ^*c*^*NA* Not active, ^*d*^Selectivity Index (SI) = CC_50_/IC_50_.

In the attempt to further evaluate the spectrum of antiviral activity, we focused our attention on **Mix 1c**, which showed a simpler chromatographic profile for the following analytical study. It was initially tested against a negative-strand RNA virus, the Influenza A virus (IAV; Table 3). MDCK and A549 cell lines were used to evaluate cytotoxicity and antiviral potency against IAV. The selected mixture did not display cytotoxicity on both cell lines. In particular, **Mix 1c** showed a high selectivity index (SI) on MDCK compared to A549 cells. We further expanded our investigation to evaluate the efficacy of **Mix 1c** against SARS-CoV-2 (Table 3). While this modified prebiotic mixture exhibited moderate toxicity in Caco-2 cells, it demonstrated potent antiviral activity against the pandemic virus, with a low micromolar IC_50_ of 3.6 µg/mL. These compelling results make **Mix 1c** a promising source of broad-spectrum antiviral agents (BSAs), warranting further in-depth exploration.

**Table 3:** Cytotoxicity and antiviral activity of mixture **Mix 1c** against influenza A and SARS-CoV-2.

Cmpd.	CC_50_^a^ (MDCK)	IC_50_^b^ Influenza A	CC_50_ A549	IC_50_ Influenza A	CC_50_ Caco-2	IC_50_ SARS-CoV-2
**Mix 1c**	169.6	2.7 ± 1.6 (SI^c^ = 62.8)	16	2.5 ± 1.1 (SI = 6.4)	13.2	3.6 ± 2.0 (SI = 3.7)

^*a*^*CC*_*50*_ half-maximal cytotoxic concentration (µg/mL), ^*b*^*IC*_*50*_ half-maximal inhibitory concentration (µg/mL), ^*c*^Selectivity Index (SI) = CC_50_/IC_50_.

### Analytical studies and antiviral screening of sub-fractions

**Fig. 2:**
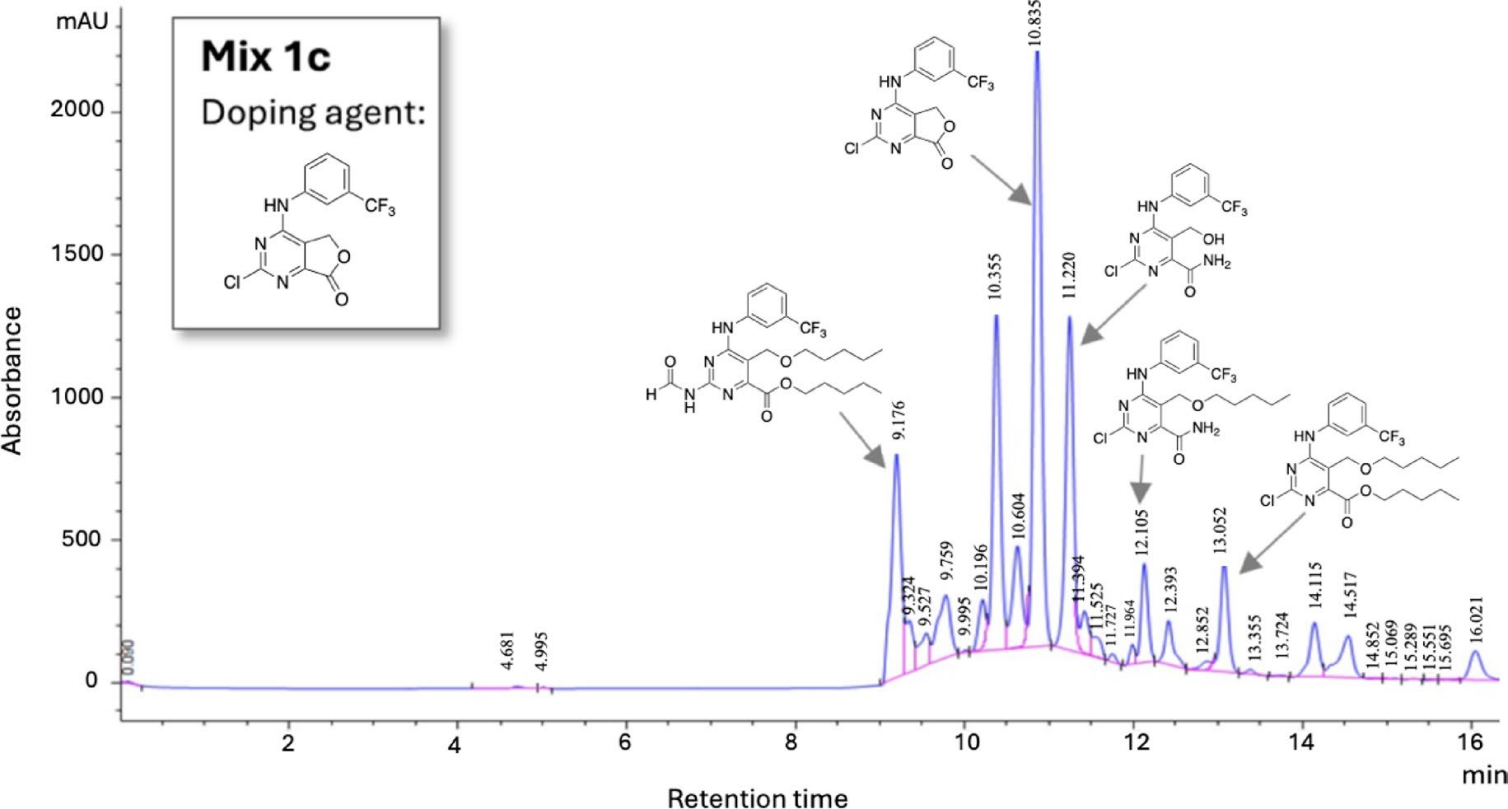
Chromatogram of **Mix 1c** showing the structure of main components by partial deconvolution. Separation was performed on a Zorbax Eclipse XDB-C18 column (250 × 4.6 mm, 5 μm) at a flow rate of 0.6 mL/min. Eluent A: H₂O^+^ (0.1% formic acid); eluent B: ACN/MeOH 1:1 (v/v). Gradient: 0–2 min, 0% B; 2–16 min, to 98% B; 16–20 min, 98% B; 20–21 min, return to 0% B. Detection: UV at 254 nm. MSD in dual mode. The doping agent used to generate **Mix 1c** is shown in the top left corner.

Next, an assessment of the stability of the complex **Mix 1c** was conducted: the sample was kept in solution (either CH_3_CN or DMSO) at room temperature for more than a month. The resulting chromatographic profile was compared with the profile of a freshly prepared solution of **Mix 1c**. This analysis revealed no significant qualitative and quantitative differences between the two profiles, demonstrating the robust stability of the **Mix 1c** (Fig. S1, Supplementary Material). The synthetic reproducibility of **Mix 1c** was also assessed, with three independent reactions yielding identical chromatographic profiles (Fig. S2, Supplementary Material). This important finding underscores the potential of **Mix 1c** as a strong candidate for further in-depth evaluation. The LC-UV/MS analysis supported by the knowledge of the chemical reactivity of the doping agent **1**^27,28^, facilitated the exploration of the complex mixture **Mix 1c**. The employed reaction conditions led mainly to the opening of the lactone ring of the “doping agent” **1**, followed by the conversion into the corresponding pentyl ether derivatives. Interestingly, C2 substitution appeared to be more challenging than lactone ring opening, as evidenced by the identification of only one C2-substituted compound, compared to the numerous compounds characterized by an opened lactone ring. This enabled a partial deconvolution of the complex mixture **Mix 1c**, ultimately leading to the identification of its main components (Fig. 2).

**Fig. 3:**
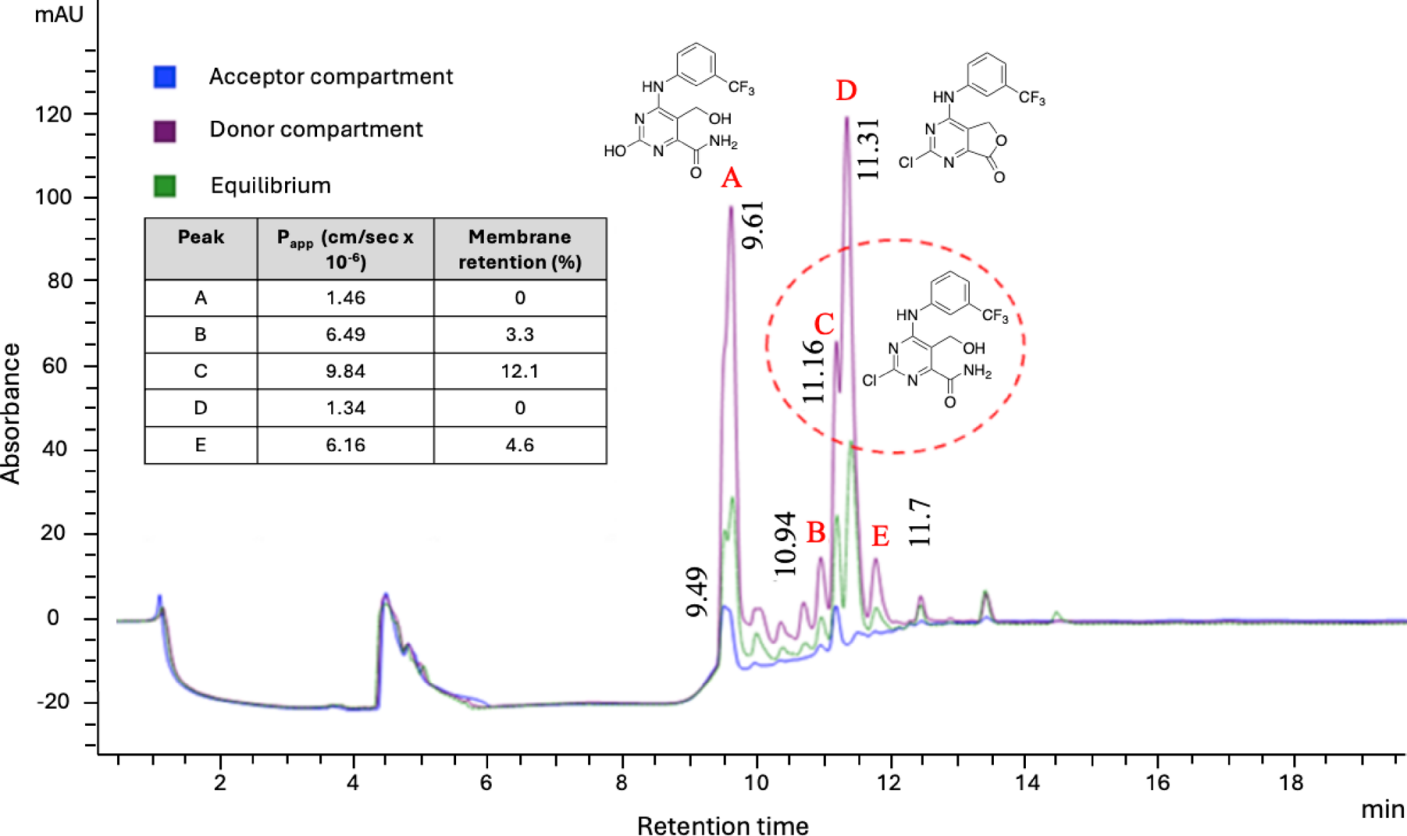
Overlaid chromatograms (254 nm) from PAMPA assay showing the composition of donator compartment (violet), acceptor compartment (blue) and equilibrium (green) over time. The analysis was performed by LC-UV/MS on a Zorbax Eclipse XDB-C18 column (250 × 4.6 mm, 5 μm) using a binary gradient of H₂O^+^ (0.1% formic acid) and ACN/MeOH (1:1, v/v) at a flow rate of 0.6 mL/min; structures of the main components obtained by partial deconvolution are shown; Donor Compartment represents the remaining compound in the donor side at the end of the experiment; Acceptor Compartment represents compounds that permeated through the artificial membrane into the acceptor side; Equilibrium represents the equilibrium concentration where donor and acceptor compartments would reach equal concentrations. Insert table on the left shows P_app_ values and membrane retention expressed as percentage of compound unable to reach the acceptor compartment for peaks A-E.

**Scheme 1:**
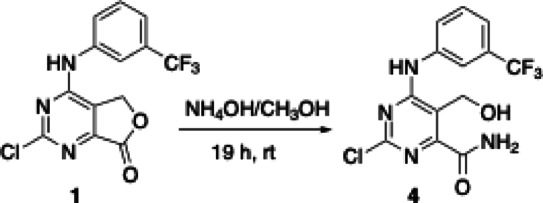
Synthesis of compound **4**. Details are reported in the methods section.

To get further insights on which components of the mixture could be responsible for the observed antiviral activity in cells, **Mix 1c** was analyzed using the parallel artificial membrane permeability assay (PAMPA). This assay simulates the passive cellular uptake and helps pinpoint molecules capable of crossing the phospholipid membrane, potentially enabling them to exert antiviral activity. **Mix 1c** was thus solubilized in 1:1 DMSO: PBS 10 mM pH 7.4 at a concentration of 0.5 mM and incubated for 5 h. After this time, the passive permeability of substances from a donor compartment into an acceptor compartment was determined, and the variation of the composition of the whole mixture was evaluated. As shown in Fig. 3, the compound corresponding to peak C exhibited higher passive permeability (Papp = 9.84 × 10^−6^ cm/sec), making it one of the most likely contributors to the antiviral activity of the mixture. Due to the analytical complexity of isolating and characterizing all major components of **Mix 1c**, we initially prioritized the detailed characterization of the most permeable component (peak C), whose structure was hypothesized through partial deconvolution studies. This hypothetical structure (peak C, Fig. 2) was easily synthesized by stirring the “doping agent” **1** in a solution of NH_4_OH in methanol for 19 h at room temperature (Scheme 1).

**Fig. 4:**
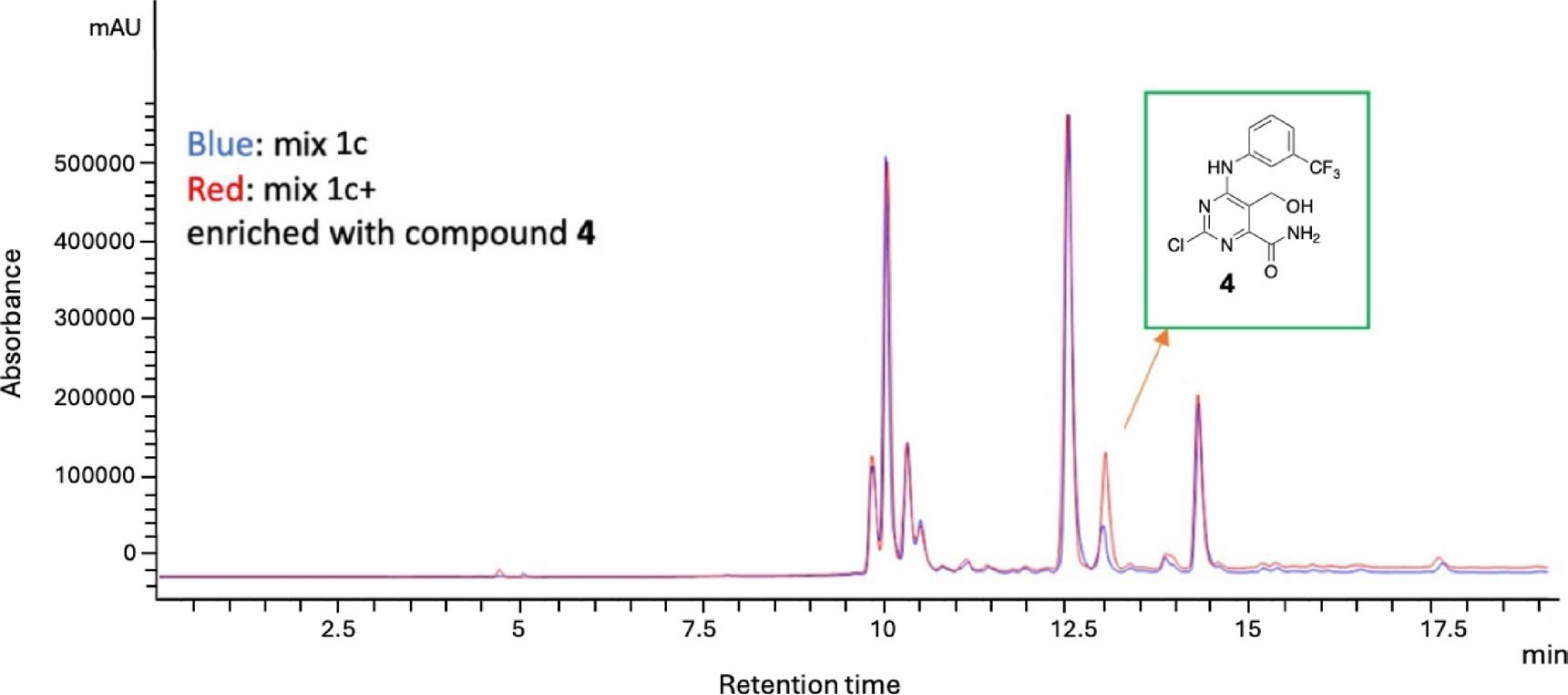
Overlaid chromatograms of **Mix 1c** (blue) and **Mix 1c+** (Red) enriched with the synthesized compound **4**, corresponding to peak C in Fig. 3. Chromatogram spectra were acquired at 254 nm. The chromatographic parameters were the same used for PAMPA assay. The chromatographic column was Zorbax Eclipse XDB-C18 column (250 × 4.6 mm, 5 μm) using the binary gradient H₂O^+^ (0.1% formic acid) and ACN/MeOH (1:1_v/v_) and a flow rate of 0.6 mL/min.

This approach allowed us to use the synthesized compound **4** as a standard to determine its presence in the **Mix 1c** using the standard addition method. We enriched **Mix 1c** (referred to as **Mix 1c+**) with a known quantity of compound **4**, and then performed chromatographic analysis to compare the spectrum of the enriched mixture with that of the original **Mix 1c.** As evident from the overlay of two chromatograms presented in Fig. 4, compound **4** aligned precisely with the peak attributed to the more permeable component of the original **Mix 1c**. Once confirmed that compound **4** was the most permeable component of **Mix 1c**, it underwent to the same screening workflow applied to prebiotic mixtures, testing it against DENV, WNV, HIV-1, and SARS-CoV-2. Unfortunately, despite the absence of significant toxicity across diverse cell lines (CC_50_ > 200 µM), compound **4** was devoid of any antiviral activity against all viruses that were inhibited by the full **Mix 1c** (Table S1, Supplementary Material).

**Fig. 5:**
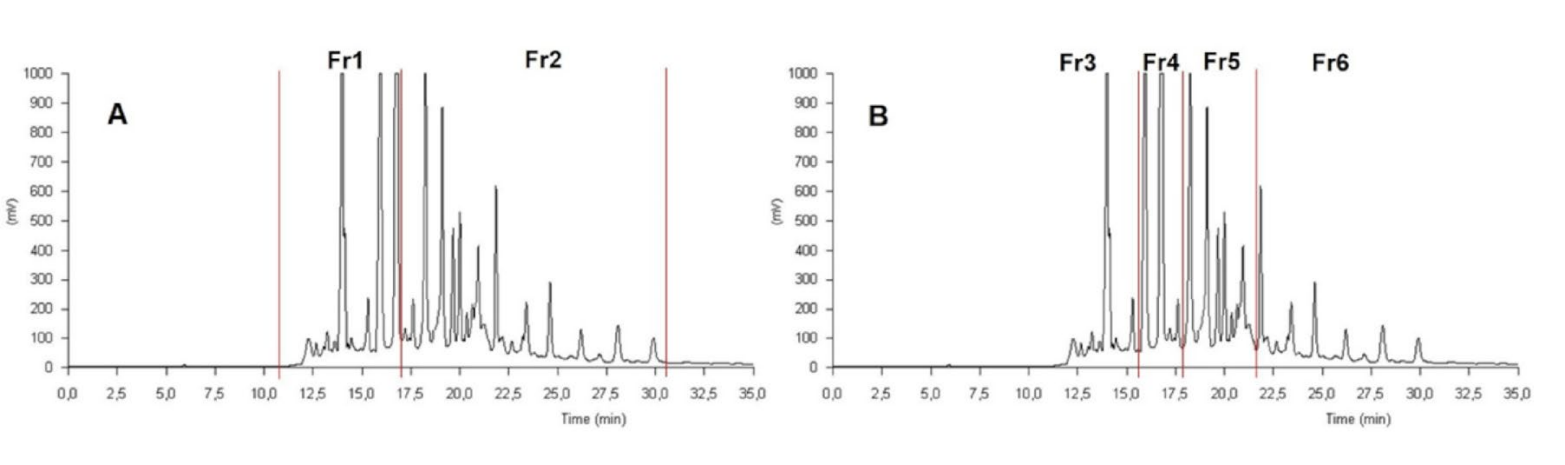
Chromatographic overview of (**A**) first separation of **Mix 1c** in two sub-mixtures (Fr1 and Fr2); and (**B**) second separation of **Mix 1c** in four sub-mixtures (Fr3 through Fr6). The separation was performed on a Zorbax Eclipse XDB-C18 column (250 × 9.4 mm, 5 μm) using a gradient of ACN and H₂O (both with 0.1% formic acid) at 2 mL/min. The sample was dissolved in ACN/MeOH (1:1,v/v, 5 mg/mL), and 200 µL were injected. The gradient started at 0% B for 4 min, increased to 98% in 25 min, and was held at 98% for 10 min. Chromatogram spectra were acquired at 254 nm. The collection of fractions were done at specific times: in the first separation (Fig. 5A) Fr1 was collected from minute 11 until 17; Fr2 from minute 17 until 30.8; in the second separation (Fig. 5B) the fractions were collected from minute 0 to 15.7 (Fr3), from minute 15.7 to 17.8 (Fr4), from minute 17.8 to 22 (Fr5), from minute 22 to 35 (Fr6).

As an alternative strategy to facilitate the identification of active components within the prebiotic **Mix 1c**, the mixture was subjected to chromatographic separation into simpler sub-fractions, which were then individually tested for antiviral activity. We initially separated the original mixture (**Mix 1c**) into two sub-fractions (Fr1, Fr2), and subsequently fractionated the same **Mix 1c** further into four sub-fractions (Fr3 through Fr6). Figure 5 visually presents the sub-fractions derived from the original **Mix 1c**. Using the chromatographic conditions reported in Materials & Methods, the sub-fractions were collected using the appropriate semi-preparative column and then analyzed with a UV detector, providing distinctive profile for each sub-fractions. Each sub-fraction was then dried and submitted to biological evaluation. Most strikingly, none of the simpler sub-fractions showed any activity against WNV, DENV, HIV, and SARS-CoV-2. Even more interestingly, when Fr3-6 were recombined (forming **Mix 1c-R**) to reconstruct the original prebiotic mixture, the broad-spectrum antiviral activity was restored, albeit with a reduction in efficacy against SARS-CoV-2 (Table 4).

**Table 4:** Cytotoxicity and antiviral activity of sub-fractions (Fr1-Fr6) obtained from different separations of the **Mix 1c**. ^a^CC_50_ half-maximal cytotoxic concentratio﻿n (µg/mL), ^b^IC_50_ half-maximal inhibitory concentration (µg/mL), ^c^NA not active, ^d^ND not determined.

Cmpd.	CC_5_^a^ (Huh7)	IC_50_^b^ WNV	IC_50_ DENV	CC_50_ (H9)	IC_50_ HIV	CC_50_ (Caco-2)	IC_50_ SARS-CoV-2
**Fr1**	15.2	NA^c^	NA	33.6	NA	ND^d^	ND
**Fr2**	3.6	NA	NA	18.4	NA	ND	ND
**Fr3**	76	NA	NA	40	NA	68	NA
**Fr4**	38	NA	NA	< 40	NA	30	NA
**Fr5**	10	NA	NA	16	NA	22	NA
**Fr6**	28	NA	NA	33.2	NA	22	NA
**Mix 1c-R**	32.9	3.6 ± 2.7 (SI = 9.1)	3.1 ± 1.7 (SI = 10.6)	40	NA	19.1	NA
**Mix 1c**	64	3.3 ± 1.4 (SI = 19.5)	7.2 ± 2.7 (SI = 8.8)	26	NA	13.2	3.6 ± 2.0 (SI = 17.8)

This partial reduction in activity could be due to the loss of some labile or volatile compounds during the previous drying process of the sub-fractions that were used to reconstruct **Mix 1c-R**. Despite being diminished, the recovered antiviral activity of the reconstructed mixture underscores the intricate synergy among the components of the non-natural **Mix 1c**, confirming its overall antiviral potential.

A key challenge in characterizing complex mixtures such as **Mix 1c** lies in the identification of individual active compounds responsible for the observed antiviral effects. While the failure to isolate a single active component highlights the potential importance of synergistic interactions between poorly active molecules within the mixture, it also raises the possibility that low-abundance compounds contribute significantly to the activity but might be labile, volatile and remain below the detection or isolation threshold using standard methods. A more extensive deconvolution efforts should be prioritized once additional mixtures or sub-fractions, exhibiting measurable and reproducible antiviral activity at the individual fraction level, are identified. This will enable targeted characterization of more potent or higher-concentration active components and facilitate a more focused application of advanced analytical techniques (such as high-resolution LC-MS/MS) to unravel the molecular basis of the antiviral activity. Until such active fractions are found, the current evidence supports the concept that chemical complexity and synergy within evolution-inspired mixtures represent a promising, albeit challenging, avenue for antiviral discovery beyond traditional single-compound paradigms.

## Discussion

In this study, we have modified the well-established formamide-based prebiotic model, introducing specific doping agents to drive the synthesis of unconventional, non-natural molecules that would not typically emerge through natural evolutionary processes. These novel compounds might have the potential to exhibit broad-spectrum antiviral activity while remaining well tolerated by the host’s defence system. Indeed, a few of these non-natural prebiotic mixtures showed low cytotoxicity against different cell lines and were also endowed with a promising antiviral activity against different viruses. However, isolated sub-fractions or components from the most promising antiviral mixture (**Mix 1c**) did not show any antiviral properties. While unexpected, this phenomenon (active mixture vs. inactive components) is consistent with experimental observations in certain natural extracts, where biological activity often arises from the synergistic interplay of multiple constituents rather than from a single compound^29,30^. The combined activity of different components frequently surpasses what any single element can achieve on its own and, sometime, is the only way to achieve a biological effect from a natural/botanical extract^31^. The results obtained from **Mix 1c** suggest that, in this case, no single compound is solely responsible for the observed antiviral activity. This may be due to the low potency of individual components, which only become biologically effective through synergistic interactions within the mixture. Notably, the broad-spectrum antiviral activity was restored—though partially—when the isolated sub-fractions were recombined (forming **Mix 1c-R**) to reconstruct the original **Mix 1c**. This finding highlights the importance of the collective behavior of the mixture and the intricate interplay between components that, individually, may exert minimal or no detectable activity. It is also worth considering that such synergy likely plays a dual role: while it can amplify antiviral effects, it may also contribute to cytotoxicity. Even compounds that are only mildly cytotoxic in isolation could interact in ways that result in additive or synergistic toxicity, ultimately lowering the selectivity index (SI). This explains the modest SI observed for **Mix 1c** against SARS-CoV-2 (SI = 3.7), and highlights the importance of future fractionation, deconvolution, purification and optimization work (e.g., different doping agents, solvents and reaction parameters) aimed at removing toxic or inactive molecules while enriching for more potent, better-tolerated compounds. Such refinement could eventually lead to the identification of discrete antiviral agents with improved efficacy and selectivity, enabling a more targeted exploration of this evolution-inspired chemical space.

Alternatively, these findings could be analyzed through the lens of complexity, emphasizing the functional significance of interactions between different complex mixtures and a biological system, such as virus-infected cells. Previous work by Cronin et al. has demonstrated that varying environmental conditions can give rise to distinct functional outcomes from complex chemical systems^32^. In line with this, our work suggests that specific pharmacological properties may arise from different chemical mixtures generated under non-natural prebiotic conditions. These results offer a fresh perspective on the role of complexity at the interface between chemistry and biology, one that transcends the traditional, reductionist model in which a single molecule modulates a biological function through a highly specific interaction. Instead, they support a more system-level view, where functional properties result from the collective behavior of multiple chemical and biological components.

From an evolutionary perspective, prebiotic complexity may have played a pivotal role in shaping the survival and selection of certain viral species during their co-evolution with the earliest eukaryotic cells. Our work invites intriguing speculation about the evolutionary interplay between viruses, their hosts, and the primordial biosphere. It is conceivable that specific molecular compositions within the chemically complex prebiotic environment may have influenced early interactions between ancestral viruses and emerging eukaryotic systems. This co-evolutionary dynamic could have been a key driver in the early diversification of both viral and cellular life, with complexity serving as a selective pressure that influenced which entities thrived in the face of environmental and biological challenges. In addition, by analogy with the evolutionary process that led to the development of secondary metabolites in plants as a defense mechanism against external threats, the intentional modification of a prebiotic chemistry model in the laboratory may serve as a form of “directed chemical evolution” to quickly select non-natural mixture that can help protect eukaryotic cells from viral infection. These mixtures, like phytocomplexes present in plant hydroalcoholic extracts, might represent a source of innovative antiviral agents. While our work is not intended to replicate prebiotic evolution in a literal sense, it conceptually draws upon key principles of natural evolutionary processes—namely, the generation of chemical diversity and complexity.

We acknowledge that the present study introduces a conceptual framework rather than a fully elucidated mechanism. Unlike the conventional target-based or phenotypic drug-discovery approaches focused on individual molecules, our evolution-inspired strategy embraces chemical complexity and explores underrepresented areas of chemical space. Despite its early-stage nature, this approach highlights the untapped potential of leveraging molecular diversity and mixture-based systems in antiviral discovery. By integrating perspectives from chemistry, virology, and molecular biology, this framework may be further refined to yield novel, robust antiviral agents operating outside traditional paradigms.

## Methods

### Synthetic chemistry

#### General info

All commercially available chemicals were purchased from Fisher Scientifics, Fluorochem, Merck Life Science, Sigma-Aldrich, Carlo Erba, Acros Organics, TCI and, unless otherwise noted, used without any previous purification. Solvents used for work-up and purification procedures were of technical grade. TLC was carried out using Sigma-Aldrich TLC plates (silica gel on Al foils, SUPELCO Analytical, Merck 60 F254 silica plates). Where indicated, products were purified by silica gel flash chromatography on columns packed with Merck Geduran Si 60 (40–63 μm), or Merck 60 silica gel, 230–400 mesh. Low resolution mass spectrometry measurements were performed on an Agilent 6100 Series InfinityLab LC/MSD iQ, Single Quadropole analyzer and are reported in the form of (m/z).

#### Microwave irradiation experiments

Microwave reactions were conducted using a CEM Discover Synthesis Unit (CEM Corp., Matthews, NC). The machine consists of a continuous focused microwave power delivery system with an operator-selectable power output from 0 to 300 W. The temperature inside the reaction vessel was monitored using a calibrated infrared temperature control mounted under the reaction vessel. All experiments were performed using a stirring option whereby the reaction mixtures were stirred by means of a rotating magnetic plate located below the floor of the microwave cavity and a Teflon-coated magnetic stir bar in the vessel.

#### General procedure for the synthesis of non-natural prebiotic mixtures

In a microwave sealed tube, the opportune “doping agent” from **1–3** (0.16 mmol) was suspended in: A) pure pentanol (> 99.0%) (1.5 mL) for mixtures **Mix 1c**, **Mix 2c**, **Mix 3b** and **Mix 3c**; B) water (1.5 mL) for **Mix 1a** and **Mix 2a**; C) H_2_O/n-pentanol (> 99.0%) (1 mL:1 mL) for **Mix 1b**, **Mix 2b**, **and Mix 3a**; D) formamide, 99+% (1 mL) for **Mix 1d**, **Mix 2d**, **Mix 3d**. Formamide, 99+% (10 eq) was then added to the previous mixtures A-C. The tubes were heated at 180 °C under microwave irradiation as follows: i) 5 min for **Mix 1a**, **Mix 1d**, **Mix 2a**, **Mix 2d**, **Mix 3a**, and **Mix 3d**; ii) for 15’ for **Mix 1b**, and **Mix 2b**; iii) for 30’ for **Mix 3b**; and iv) 1 h for **Mix 1c**, **Mix 2c** and **Mix 3c**.

After this time, H_2_O and EtOAc were added and the organic phases were washed with H_2_O (x5) and brine (x3). The organic layers were dried over Na_2_SO_4_ and concentrated under vacuum. The resulting crude mixture was suspended in a 90:10 EtOH/H_2_O solution, filtering off the eventual precipitate. The hydroalcoholic solution was finally evaporated under reduced pressure to obtain the desired prebiotic mixtures.

#### Synthesis of 2-chloro-5-(hydroxymethyl)−6-((3-(trifluoromethyl)phenyl)amino)pyrimidine-4-carboxamide (**4**)

Doping agent **1** (0.16 mmol) was dissolved in MeOH (1mL). A solution of NH_4_OH (1 mL) was added and the reaction mixture was stirred at r.t. for 19 h. After checking with TLC the end of the starting materials, the solvents were evaporated and the mixture was extracted with EtOAc. The combined organic layers were washed with brine, dried over anhydrous Na_2_SO_4_, filtered and concentrated. The crude material was purified by silica gel chromatography, using 99:1 DCM: MeOH as eluent. Yield: 53%; MS (ESI) [M + H]^+^: 347.05 m/z. ^1^H NMR **(**CDCl_3_ + CD_3_OD, 400 MHz): *δ* 5.12 (s, 2 H); 6.13 (bs, 1 H); 7.40 (d, 1 H, J = 8 Hz); 7.51 (t, 1 H, J = 8 Hz); 7.71 (bs, 2 H); 7.88 (s, 1 H); 7.90 (s, 1 H); 9.28 (bs, 1 H).

### Analysis of non-natural prebiotic mixtures

#### HPLC/UV-MS method

LC chromatographic analyses were performed by UV/LC-MS with an Agilent 1100 LC/MSD VL system (G1946C) (Agilent Technologies, Palo Alto, CA) equipped with a vacuum solvent degassing unit, a binary high-pressure gradient pump, an 1100 series UV detector, and a 1100 MSD model VL benchtop mass spectrometer. The chromatographic analyses were obtained using an analytical Zorbax Eclipse XDB-C18 column (Agilent) (250 × 4.6 mm and 5 μm of particle size). The gradient elution involved the use of a binary solution with an eluent A: H_2_O acidified with formic acid (FA) 0.1%_v/v_, and an eluent B: ACN/MeOH 1:1_v/v_, and the analyses were performed with a flow rate of 0.6 mL/min and an injection volume of 5 µL at room temperature. The analysis started with 0% of B (from t = 0 to t = 2 min), then B was increased to 98% (from t = 2 to t = 16 min), then kept at 98% (from 16 to 20 min) and finally returned to 0% of eluent A in one minute. The MSD worked in dual mode and the UV detector operated at 254 nm. The ESI-MS parameters were: capillary voltage 1000 V, drying gas 5 L/min, drying gas temperature 300.0 °C, nebulizing gas 60.0 psi and vaporizing temperature 200 °C. Nitrogen was used as nebulizer gas and drying gas.

#### Stability studies

Stability in DMSO and ACN was conducted for **Mix 1c** at room temperature, protected from light, for more than 30 days analyzing the solutions (1 mg/mL) once a week in the above reported chromatographic condition. The comparison was made by comparing the relative chromatograms obtained at 254 nm (See Fig. S2, Supplementary Material).

#### Parallel artificial membrane permeability assay (PAMPA)

In order to assess the apparent permeability of various components of the mixture, a stock solution in DMSO of **Mix 1c** was prepared at the final concentration of 1 mM. By diluting the stocks 1:1 v/v with phosphate buffer (PBS 25 mM, pH 7.4), donor solutions were made. To mimic the gastrointestinal (GI) phospholipidic bilayer, 10 µL of a 1% w/v dodecane solution of phosphatidylcholine (PC) was used to coat filters. The acceptor solution, made of 1:1 v/v DMSO/PBS, was added to each well (300 µL), while the donor solution (150 µL) was added to each well of the filter plate. The sandwich plates were assembled and incubated for 5 h at room temperature. At the time point, the plates were separated, and the amount of compound passed through the phospholipid bilayer was measured by UV/LC-MS. Finally, apparent permeability (Papp) and membrane retention (MR%) were calculated for the main components.

#### Standard addition method

**Mix 1c** was enriched with the synthesized compound **4**, and the chromatogram was acquired at 254 nm using a Zorbax Eclipse XDB-C18 column (250 × 4.6 mm, 5 μm) with a binary gradient H₂O^+^ (0.1% formic acid) and ACN/MeOH (1:1_v/v_) and a flow rate of 0.6 mL/min.

#### Chromatographic separation

Fractionation of **Mix 1c** was performed on a Varian ProStar apparatus equipped with two pumps and a UV detector using a semipreparative Zorbax Eclipse XDB-C18 column (Agilent) (250 × 9.4 mm and 5 μm of particle size). The mixture was dissolved in ACN/MeOH 1:1_v/v_ (5 mg/mL) and 200 µL injected each time. The separation was performed by using the same solvents as in the HPLC-UV-MS method at 2 mL/min and a modified gradient: 0% of B for 4 min then % of B was increased to 98% in 25 min, then kept at 98% for 10 min. In the first separation (Fig. 5A), Fr1 was collected from minute 11 until 17; Fr2 from minute 17 until 30.8. In in the second separation (Fig. 5B) the fractions were collected from minute 0 to 15.7 (Fr3), from minute 15.7 to 17.8 (Fr4), from minute 17.8 to 22 (Fr5), from minute 22 to 35 (Fr6). The collected fractions were dried under nitrogen flow, weighted, and submitted to biological evaluation after a chromatographic control.

## Biology

### Viruses and cells

The New Guinea C DENV serotype 2 and the WNV lineage 1 (Italy/2009) strains were kindly provided by the Istituto Superiore di Sanità (Rome, Italy) while the SARS-CoV-2 strain, belonging to lineage B1 (EPI_ISL_2472896) was kindly provided by the Department of Biomedical and Clinical Sciences Luigi Sacco, University of Milan. Once expanded in VERO E6 (African green monkey kidney cell line, ATCC catalog. n. CRL-1586), DENV, WNV and SARS-CoV-2 viral stocks were stored at − 80 °C and titrated as previously described^33^. HIV-1 wild-type reference strain NL4-3 (catalog. n. ARP2006) was obtained through the NIH AIDS Reagent Program and the viral titer was calculated in TZM-bl cells through the detection of β-galactosidase expression. Cell-based assays were carried out on the Huh7 hepatocarcinoma cell line (kindly provided by Istituto Toscano Tumori, Core Research Laboratory, Siena, Italy), on the Caco-2 adenocarcinoma colorectal cell line (ATCC catalog. n. HTB-37), on the suspension H9 cell line (repository code ARP0001, NIBSC Centre for AIDS reagents) and on the adherent TZM-bl cell line (repository code ARP5011, NIBSC Centre for AIDS reagents). Huh7 and Caco-2 were used to determine the cytotoxicity and the antiviral activity of candidate compounds against flaviviruses and SARS-CoV-2, respectively. H9 cells in combination with TZM-bl cells were used to evaluate the compounds against HIV-1, as described in the antiviral assays section. High glucose Dulbecco’s Modified Eagle’s Medium with sodium pyruvate and L-glutamine (DMEM; Euroclone) was used to grow Huh-7 and TZM-bl cell lines. Minimum Essential Eagle Medium (EMEM; Euroclone) was used to propagate Caco-2 cell line. All media were supplemented with 10% fetal bovine serum (FBS; Euroclone) and 1% penicillin/streptomycin (Pen/Strep, Euroclone). The same medium with a lower concentration of FBS (1%) was used for viral propagation, cytotoxic and antiviral experiments in adherent cell lines. H9 cell lines were grown, propagated and infected in Roswell Park Memorial Institute medium (RPMI-1640, Sigma) supplemented with 10% fetal bovine serum (FBS; Euroclone), 2 mM L-glutamine and 1% penicillin/streptomycin (Pen/Strep, Euroclone). All cell lines were grown at 37 °C in a 5% CO_2_ atmosphere in a humidified incubator. A549 (ATCC catalogue no. CCL-185) and MDCK (ATCC catalogue no. CCL-34) were used to determine the cytotoxicity and anti-influenza activity of candidate compounds. Allantoic cavities of 11-day-old embryonated chicken eggs were used to grow influenza viruses A/Puerto Rico/8/34 H1N1 (PR8 H1N1). Viral suspension was inoculated in the allantoic cavity and incubated for 48 h at 37 °C, then infected eggs were maintained overnight at 4 °C. Subsequently, the allantoic fluid was collected and clarified by centrifugation (2,500 x g for 30 min). The recovered virus was used for the infection of MDCK and A549 cells. Allantoic fluid from uninfected eggs was used as reference for mock infection^34^.

### Cytotoxicity assay

The cytotoxicity of investigational compounds was determined by CellTiter-Glo 2.0 Luminescent Cell Viability Assay (Promega) in the cells used to subsequently test flaviviruses, SARS-COV-2 and HIV-1 (Huh7, Caco-2) as previously described^35^. Cells were treated in duplicate with decreasing concentrations of investigational compounds diluted to maintain the final DMSO concentration below 0.5% v/v. The luminescence values obtained from cells treated with investigational compounds or DMSO control were measured through the GloMax^®^ Discover Multimode Microplate Reader (Promega) and elaborated with GraphPad PRISM software version 9.0 (La Jolla, San Diego, CA, USA). The half-maximal cytotoxic concentration (CC50) was calculated using a non-linear regression analysis of the dose–response curves and the E-Canything GraphPad function. Untreated and death cells were used to normalize the 100% and 0% of viability, respectively. The cytotoxicity assay of candidate compounds was evaluated on cell lines used for influenza virus infection by the MTT [3-(4,5-dimethylthiazol-2-yl)−2,5-diphenyltetrazolium bromide] assay. Briefly, cells were seeded in 96-well plates at a density of 2 × 10^4^ cells/well in 100 µL of complete RPMI without phenol red for 24 h at 37 °C. Subsequently, cell monolayers were treated or not with increasing concentrations (0.5, 2.5, 5, 10 and 20 µM) of compounds for 24 h at 37 °C. After 24 h, 10 µL of MTT solution (5 mg/mL) were added to each well for 3–4 h at 37 °C. Afterwards, each sample was acidified by adding 0.1 N HCl in isopropanol (100 µL/well) for 30 min in slow agitation to ensure that all formazan crystals were dissolved. Absorbance of samples was read at 570 nm, using an automatic plate reader (Multiskan EX, Ascent Software, Thermo Fisher Scientific). Untreated cells were used as control. The 50% cytotoxic concentration (CC_50_), defined as the compound’s concentration required to reduce cell viability by 50%, was calculated using the GraphPad, Prism 6 Software.

### Antiviral assays on flaviviruses and SARS-CoV-2

To determine the antiviral activity of candidate compounds against DENV, WNV and SARS-CoV-2, a direct yield reduction assay based on the infection of cells in the presence of serial drug dilutions was performed as previously described with minor modifications^35^. Briefly, Huh7 or Caco-2 cells, pre-seeded in 96-well format, were infected with DENV and WNV viral stocks at 0.005 multiplicity of infection (MOI) or with SARS-CoV-2 at 0.01 MOI. After 1 h of adsorption of the virus at 37 °C, viral inoculum was removed and serial dilutions of each tested compound, starting from the not-toxic dose, were added to the infected cells. After 48 h of incubation for WNV and 72 h for DENV and SARS-CoV-2, the antiviral activity was measured on the cell monolayer using immunodetection assay (IA), as previously described [Ref. 35]. Absorbance was measured at 450 nm optical density (OD450) using the Absorbance Module of the GloMax^®^ Discover Multimode Microplate Reader (Promega). In each plate the suitable reference compound, a mock control (uninfected cells) and a virus control were included. Each IA run was validated when the OD450 values of virus control showed an OD450 > 1. All drug concentrations were tested in duplicate in two independent experiments. In each plate, Sofosbuvir (MCE^®^ cat. HY-15005) and Remdesivir (MCE^®^ cat. HY-104077), purchased from MedChem Express (http://www.medchemexpress.com) were used as reference compounds against flaviviruses and SARS-CoV-2, respectively. Infected and uninfected cells without drugs were used to calculate the 100% and the 0% of viral replication, respectively. The half-maximal inhibitory concentration (IC_50_) was calculated through a non-linear regression analysis of the dose–response curves generated with GraphPad PRISM software version 9 (La Jolla, CA, USA). The Selectivity Index (SI) of the compounds was calculated as the ratio between the CC_50_ and the IC_50_.

### Antiviral assays on HIV-1

The antiviral activity of investigational compounds was evaluated by measuring the IC_50_ values against the HIV-1 wild-type reference strain NL4-3 in a TZM-bl cell line based phenotypic assay named BiCycle Assay^36^. This method includes a first round of infection in H9 cells, where the HIV viral stock at 0.02 MOI was mixed with the cells and added to 96-well plate containing decreasing dilution of compounds. In each plate were included the mock control (uninfected cells), the virus control and Raltegravir (code HY-10353) as reference compound. After 72 h, 50 µl of supernatants from each well were used to infect the TZM-bl cell line, which allows the quantitative analysis of HIV-1 infection by measuring the expression of the luciferase gene integrated in the genome of the cells under the control of the HIV-1 L promoter. After 48 h, dose–response curves were generated by measuring reporter gene expression in each well by using Bright-Glo Luciferase Assay (Promega) through the GloMax^®^ Discover Multimode Microplate Reader (Promega). Relative luminescence units measured in each well were elaborated with the GraphPad PRISM software version 9 to calculate IC_50_ values.

### Antiviral assays on influenza

The anti-influenza activity of candidate compounds was evaluated by Hemagglutination assay (HAU) in the supernatants of infected cells recovered after 24 h from infection^37^. Briefly, MDCK and A549 cells seeded in 12-well plates at a density of 2.5 × 10^5^ cells/well, were infected with influenza virus A/Puerto Rico/8/34 H1N1 and incubated for 1 h at 37 °C. After viral adsorption, cells monolayers were washed with PBS and then, fresh medium supplemented with 2% FBS with different concentrations (0.5, 2.5, 5, 10 and 20 µM) of selected compounds was added for 24 h at 37 °C. Each compound solved in DMSO was diluted in RPMI (the highest concentration of DMSO added in the culture medium was equal to 0.2% of final volume). As reference, infected cells were treated with DMSO alone. The IC_50_ of each compound was calculated using the GraphPad, Prism 6 Software. The SI was calculated as described above.

### Quantification and statistical analysis

Statistical analyses were performed using Prism 6 software (Graphpad Software, La Jolla, CA, USA). Differences between treated and untreated control were estimated using 2-way ANOVA followed by Dunnett’s multiple comparisons test. Values were expressed as the mean ± SD and *p* < 0.05 was considered statistically significant.

## Supplementary Information

Below is the link to the electronic supplementary material.

Supplementary Material 1

## Data Availability

The authors confirm that the data supporting the findings of this study are available within the article.
