# Supplementary material for: Harnessing prebiotic formamide chemistry: a novel platform for antiviral exploration
Source: Sci Rep. 2025 Aug 2;15:28197. doi: 10.1038/s41598-025-14001-3 (PMC12317059; doi:10.1038/s41598-025-14001-3)
Supplement: Supplementary file 1 — Supplementary Material 1 [file 41598_2025_14001_MOESM1_ESM.docx]

**Supplementary Material**

**Harnessing Prebiotic Formamide Chemistry: a Novel Platform for Antiviral Exploration**

Maria Grazia Martina,^1^ Chiara Vagaggini,^2^ Elena Dreassi,^2^ Marta De Angelis,^3,4^ Lucia Nencioni,^3^ Filippo Dragoni,^5^ Federica Giammarino,^5^ Adele Boccuto,^5^ Maurizio Zazzi,^5^ Ilaria Vicenti^5^ and Marco Radi^1,^*

^1^Dipartimento di Scienze degli Alimenti e del Farmaco, Università degli Studi di Parma, Viale delle Scienze, 27/A, 43124 Parma, Italy

^2^Department of Biotechnology, Chemistry and Pharmacy (DBCF), University of Siena, 53100 Siena, Italy

^3^Department of Public Health and Infectious Diseases, Laboratory Affiliated to Istituto Pasteur Italia-Fondazione Cenci Bolognetti, Sapienza University of Rome, P.le Aldo Moro 5, 00185 Rome, Italy

^4^Laboratory of Virology, Department of Molecular Medicine, Sapienza University, 00185 Rome, Italy

^5^Department of Medical Biotechnologies, University of Siena, Viale Bracci 16, 53100 Siena, Italy

*Correspondence: [marco.radi@unipr.it](mailto:marco.radi@unipr.it)

**Contents Page**

1. Synthetic procedures for doping agents **1-3** S2
2. **Figure S1.** Chromatographic profile of **Mix 1c** at different time-points S3-S4
3. **Figure S2.** Chromatographic profiles of three independently S5

synthesized batches **of Mix 1c**

1. **Table S1**. Cytotoxicity and antiviral activity of compound **4** S5
2. **Synthetic procedures:**

**Synthesis of Doping Agent 3**

1,5-dihydrofuro[3,4-d]pyrimidine-2,4,7(3H)-trione (2323 mg, 13.82 mmol) was suspended in freshly distilled POCl_3_ (27 mL) under N_2_ atmosphere. After adding DIPEA (3.6 mL, 20.72 mmol) to the cooled to 0 °C reaction flask, the white suspension turned dark brown. The reaction was heated at reflux for 18 hours. Volatile residues and POCl_3_ were distilled off after cooling to room temperature the reaction mixture. The crude was cooled to 0 °C and H_2_O (4 mL) was slowly added, the mixture was then stirred at room temperature until a brown solid precipitated (~1 hour), and the brown solid obtained was filtered over a Buchner funnel, and washed with cold H_2_O and Et_2_O. The waters of filtration were neutralized with NaHCO_3_ and extracted with EtOAc, washed with brine, dried over Na_2_SO_4_, filtered and evaporated under vacuum. The reaction’s crude was purified by flash chromatography using petroleum ether/EtOAc 8/2 as eluent to afford pure doping agent **3**.

2,4-dichlorofuro[3,4-d]pyrimidin-7(5H)-one (**3**): Yield: 95%. MS (ESI) [M+H]+: 204.9 m/z, [M+Na]+: 226.9 m/z. ^1^H-NMR (CDCl_3_ 400 MHz): δ 5.55 (s, 2H) ^13^C-NMR (CDCl_3_ 100 MHz): δ 67.50; 137.69; 156.84; 158.42; 160.44; 165.74.

**General procedure for the synthesis of Doping Agents 1 and 2**

In a round-bottom flask, equipped with a condenser, doping agent **3** (500 mg, 2.44 mmol, 0.5 eq.) and 0.25 eq. of the opportune aniline (3-(trifluoromethyl)aniline for doping agent **1**; sulfanilamide for doping agent **2**, were added to a pre-heated solution (85 °C) of n-BuOH (10 mL). After 20 minutes, intermediate 3 was added again (500 mg, 2.44 mmol, 0.5 eq). After 10 minutes, Et_3_N (212.5 µL, 1.52 mmol, 0.25 eq) and another portion of proper aniline (0.25 eq) were added. After 10 minutes, Et_3_N (212.5 µL, 1.52 mmol, 0.25 eq) and the proper aniline (0.25 eq) were added again. After 10 minutes, another addition of 0.25 eq of both Et_3_N and the proper aniline was done. After 1hour and 40 minutes, the reaction was over. The solvent of the reaction was evaporated under vacuum and the crude was extracted with EtOAc. The combined organic layers were washed with NH_4_Cl and then brine, dried over anhydrous Na_2_SO_4_, filtered and concentrated. The crude material was purified by flash chromatography using DCM:MeOH+HCOOH 80% aq 99:1+1% as eluent for doping agents **1** and EtOAc:petroleum ether+HCOOH 80% aq 8:2+1% as eluent for doping agents **2**.

2-chloro-4-((3-(trifluoromethyl)phenyl)amino)furo[3,4-d]pyrimidin-7(5H)-one (**1**): Yield: 53%. MS (ESI) [M+H]+: 330.0 m/z. ^1^H-NMR (DMSO-d*_6_* 400 MHz): δ 5.39 (s, 2H); 7.56 (d, 1H, J = 8 Hz); 7.70 (t, 1H, J = 8 Hz); 8.03 (d, 1H, J = 8 Hz); 8.17 (s, 1H); 10.59 (bs, 1H).

4-((2-chloro-7-oxo-5,7-dihydrofuro[3,4-d]pyrimidin-4-yl)amino)benzenesulfonamide (**2**):Yield: 50%. MS (ESI) [M+H]+: 341.2 m/z, [M+Na]+: 363.1, [M+K]+: 379.3 m/z. ^1^H-NMR (DMSO-d*_6_* 300 MHz): δ 5.93 (s, 2H); 7.35 (s, 2H); 7.88 (s, 4H); 10.60 (bs, 1H). ^13^C-NMR (DMSO-d*_6_* 100 MHz): δ 68.00; 121.68; 126.45; 127.15; 140.04; 141.15; 152.16; 157.00; 160.64; 164.20; 167.51.

1. **Supplementary figures:**


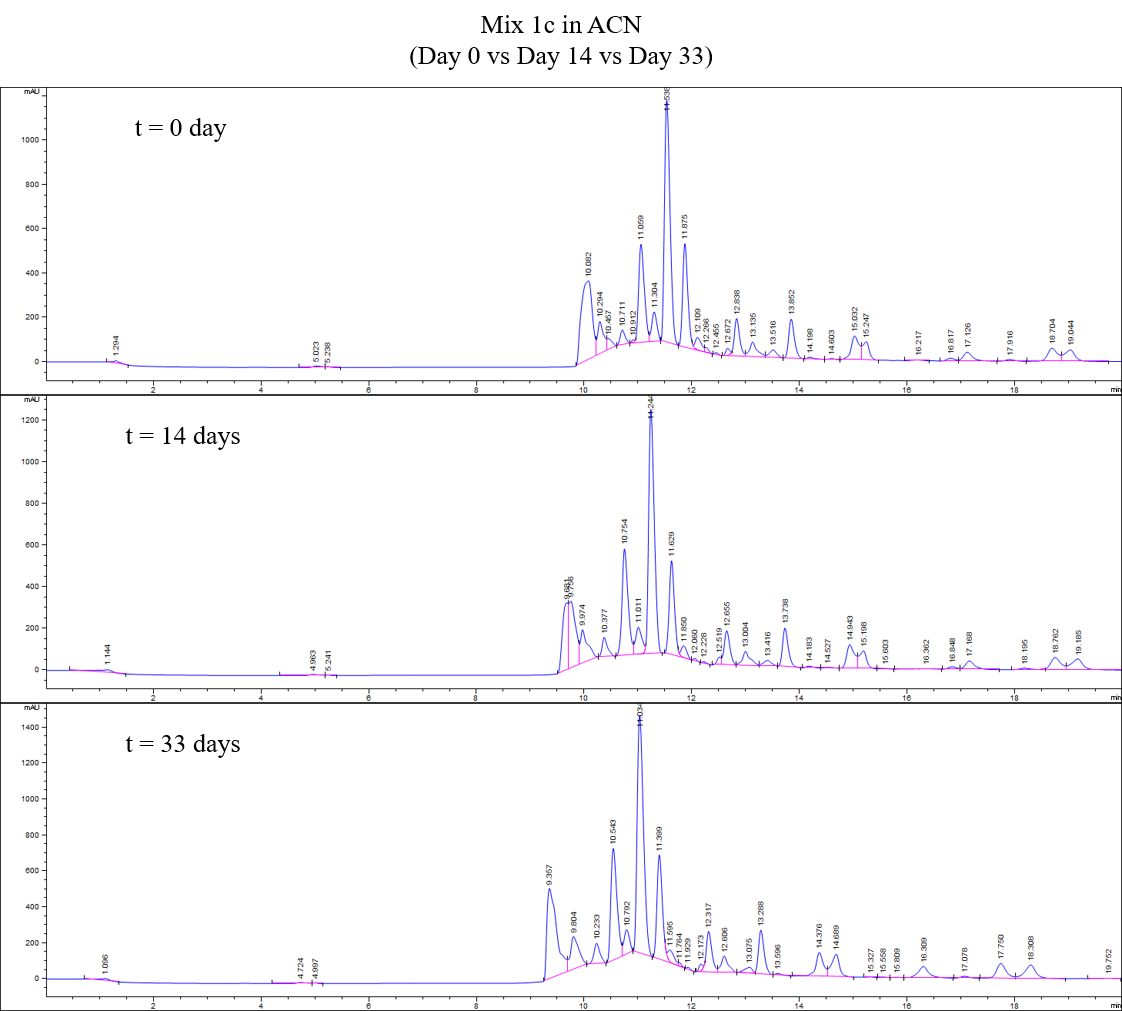


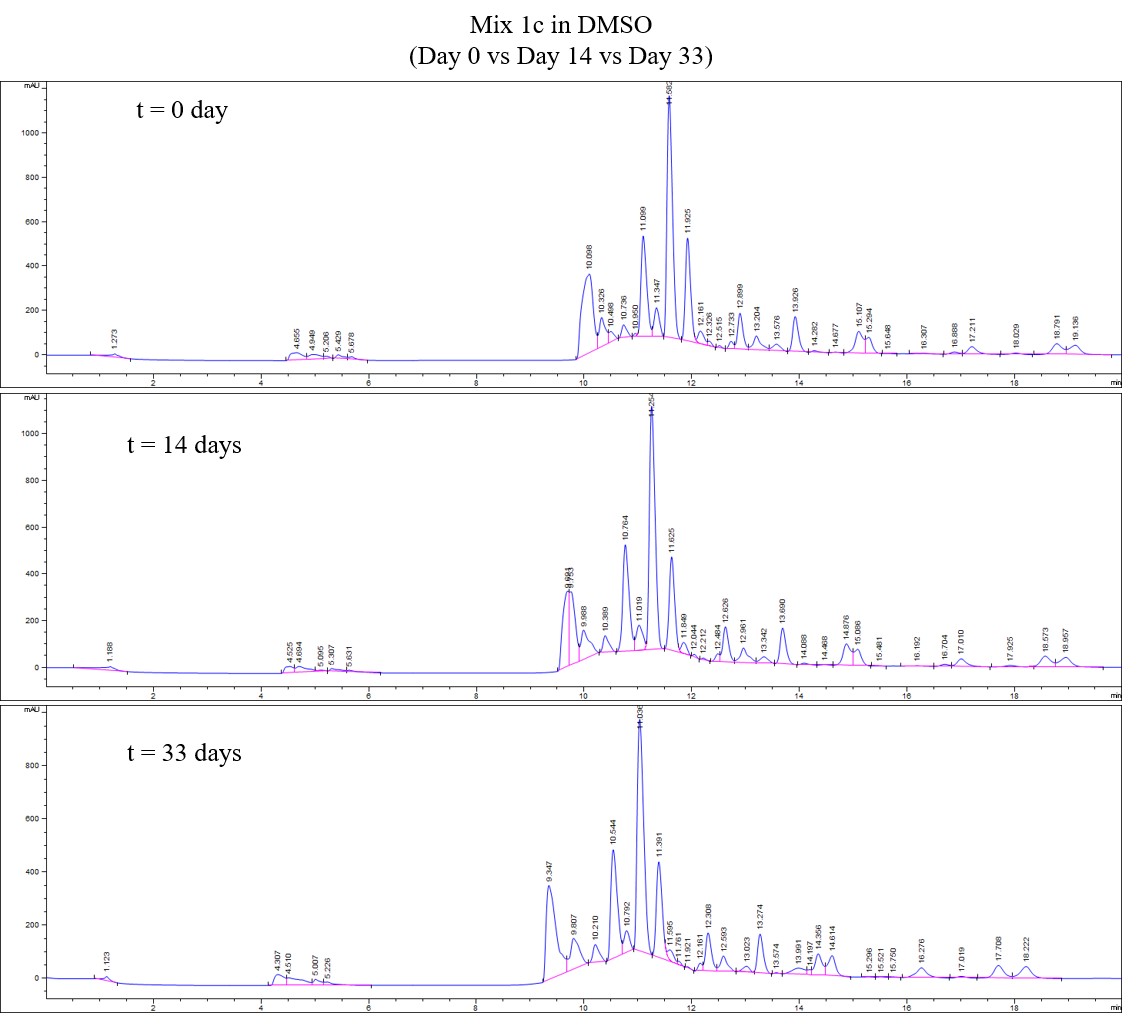


**Figure S1.** Chromatographic profile of **Mix 1c** at different time-points. Separation was performed on a Zorbax Eclipse XDB-C18 column (250 × 4.6 mm, 5 µm) at a flow rate of 0.6 mL/min. Eluent A: H₂O^+^ (0.1% formic acid); eluent B: ACN/MeOH 1:1 (v/v). Gradient: 0–2 min, 0% B; 2–16 min, to 98% B; 16–20 min, 98% B; 20–21 min, return to 0% B. Detection: UV at 254 nm. MSD in dual mode.

**Figure S2.** Chromatographic profiles of three independently synthesized **Mix 1c** batches. Overlay for comparison (blue: Batch 1; green: Batch 2; red: Batch 3). Separation was performed on a Zorbax Eclipse XDB-C18 column (250 × 4.6 mm, 5 µm) at a flow rate of 0.6 mL/min. Eluent A: H₂O^+^ (0.1% formic acid); eluent B: ACN/MeOH 1:1 (v/v). Gradient: 0–2 min, 0% B; 2–16 min, to 98% B; 16–20 min, 98% B; 20–21 min, return to 0% B. Detection: UV at 254 nm. MSD in dual mode.

**Table S1**. Cytotoxicity and antiviral activity of compound **4**

| Cmpd. | CC_50_^a^  (*Huh7*) | IC_50_ ^b^  WNV | IC_50_  DENV | CC_50_  *(H9)* | IC_50_  HIV | CC_50_  *(Caco-2)* | IC_50_  Sars-CoV-2 |
| --- | --- | --- | --- | --- | --- | --- | --- |
| 4 | >200 | NA^c^ | NA | >100 | NA | >200 | NA |

^a^CC_50_: half-maximal cytotoxic concentration (µg/mL); ^b^IC_50_: half-maximal inhibitory concentration (µg/mL); ^c^NA: not active.
